# Prize for an Essay on Epilepsy

**Published:** 1899-01

**Authors:** 


### Prize for An Essay on Epilepsy.

A prize of one hundred dollars is offered by the board of managers of Craig Colony for the best original essay bearing upon the pathology and treatment of epilepsy. The essay must be written in English, but competition is open to the world. The award will be made upon the recommendation of a committee composed of three members of the New York Neurological Society, at the annual meeting of the board of managers of Craig Colony on October 10, 1899. The successful essay becomes the property of the Craig Colony, for publication in its annual medical report. Papers submitted for the competition should be sent to Dr. Frederick Peterson, 4 West Fiftieth Street, New York City, on or before September 1, 1899.

